# A novel body coloration phenotype in *Anolis sagrei*: Implications for physiology, fitness, and predation

**DOI:** 10.1371/journal.pone.0209261

**Published:** 2018-12-31

**Authors:** Yasmeen R. Erritouni, Beth A. Reinke, Ryan Calsbeek

**Affiliations:** Department of Biological Sciences, Dartmouth College, Hanover, New Hampshire, United States of America; Clemson University, UNITED STATES

## Abstract

In animals, color signals that convey information about quality are often associated with costs linked to the expression of coloration and may therefore be honest signals of sender quality. Honest indicators are often seen in sexual signals that are used by males to advertise quality to females. Carotenoid and pterin pigments are responsible for yellow, orange, and red coloration in a variety of taxa, but can also serve important roles as antioxidants by reducing free radicals in the body. In this study, we test the effects of a novel full-bodied orange color phenotype of the brown anole, *Anolis sagrei*, on mate choice, physiology, and survival. We found no evidence that lizards expressing the orange phenotype were preferred by females. Additionally, they did not differ in immune function, running endurance, or maximum sprint speed from lizards that did not express the novel phenotype. Pigment extractions revealed that orange body coloration resulted from pterin pigments and not carotenoids. Visual models suggest that the orange phenotype is less conspicuous to bird predators than the brown phenotype and may provide an adaptive explanation for the persistence of this trait. Given its small, yet positive effect on fitness, we expect the orange color phenotype to increase in frequency in subsequent decades.

## Introduction

Bright, conspicuous color signals that communicate species identity, social status, and individual quality are ubiquitous in animals. For a signal to advertise sender quality, the signal must have some cost tied to its production and maintenance [1]. Costs of signal production come in a variety of forms, such as increased physiological stress or predation risk [2, 3] and maintain “honesty” in the population, preventing the spread of cheaters. This concept is used to explain the evolution of signals that decrease the overall fitness of the sender [4].

Carotenoids are unique among animal pigments as they can only be produced by photosynthetic organisms [5]. Animals that use carotenoids for signaling functions, unable to produce them *de novo*, must acquire them from their diet. Therefore, the amount of carotenoids available for allocation to various functions is largely limited by foraging ability or environmental availability. As a result, yellows, oranges, and reds are good candidates for honest signals of quality in a variety of taxa (in fish: [6, 7]; in birds: [8–10]; in reptiles: [11]). Pterin pigments, however, are produced *de novo* [12].

Carotenoids and pterins can function as antioxidants, which reduce oxidative stress by reacting with reactive oxygen species (ROS) and other free radicals [13, 12]. Free radicals are molecules with unpaired electrons that can damage proteins, lipids, and DNA by reacting with them chemically [5]. ROS are formed when oxygen is consumed during normal metabolic processes. However, oxidative stress due to free radical formation increases during strenuous exercise, as oxygen consumption increases [14, 15]. Antioxidants defend against tissue damage during physical exertion by reacting with these free radicals [15]. As a result, organisms with adequate antioxidant defenses can reduce oxidative damage associated with exertion for longer periods than those without [16]. High concentrations of carotenoid and/or pterin based pigments in the integument may reduce the amount of antioxidant pigments available in the muscles and other tissues, where free radicals form during physical activity [17]. This reduced ability to combat free radicals in relevant tissues may, in turn, decrease physical endurance and overall performance [18, 19].

Carotenoids and pterins also act in the immune systems of vertebrates by aiding in the prevention of tumor formation and of infection by pathogens [5, 12, 20]. White blood cells use free radicals to combat pathogens but may also be damaged by these same free radicals [20]. Carotenoids and pterins react with excess free radicals to prevent this incidental damage, making the immune system more efficient [12, 20].

Lizards in the genus *Anolis* are generally cryptic in body coloration although they express a variety of social signals using their brightly colored dewlap–an extendable, conspicuously-colored flap of skin on the neck. The dewlap functions as a sexual and agonistic signal [21]. Both male and female *A*. *sagrei* use their brightly-colored dewlaps to advertise their species identity, sexual receptivity, and individual quality [22–24].

Recently, *A*. *sagrei* lizards captured in Florida have been found to express a novel pigmentation ranging from orange to red and covering large portions of their bodies apart from the dewlap (Armando Pou, personal communication). The red, orange, and yellow colors of *A*. *sagrei* dewlaps result from carotenoid and pterin pigments [25]. Because *A*. *sagrei* are known to allocate these pigments to integumentary tissue, carotenoids and pterins may be responsible for full-bodied orange coloration. Neither pigment has been characterized in *A*. *sagrei* skin outside of the dewlap.

This novel coloration was first noted less than ten years ago, and some reports suggest that frequencies of the orange phenotype may be as high as 0.5% (Robert Cox, personal communication). As *A*. *sagrei* exist in high densities in Florida [26], 0.5% may represent a considerable number of lizards. Here we test the hypothesis that orange individuals experience positive fitness consequences that may maintain this novel coloration in wild populations.

Possible fitness benefits of orange integument may arise via female preference. Female preference for certain male phenotypic traits may evolve as a corollary to naturally-selected sensory biases [4, 27]. For example, facets of sensory systems adapted for use in predator detection, foraging, or some aspect of species recognition might later be co-opted for use in mate preference so long as males emit a signal that corresponds to the cue to which females were attuned. In the case of *A*. *sagrei*, females are predisposed to the male dewlap for species recognition. Dewlaps may also stimulate female receptivity to mating [24]. Female *A*. *sagrei* are likely attuned to the red and orange hues present in dewlaps of male conspecifics and so may respond positively to full-body orange coloration as a sexual signal.

The use of orange pigments to color integument in *A*. *sagrei* may reduce the number of molecules available for use as antioxidants or may make them less accessible for use in non-integumentary tissues [5], representing a cost that could affect the immune system [28, 29].

Yet another cost of full-bodied orange coloration may come in the form of increased conspicuousness to predators. Because *Anolis* lizards are heavily preyed upon by visual predators (e.g., birds; [30, 31]), it is likely that visually conspicuous individuals are more prone to predation. Increased susceptibility to predation has been hypothesized to maintain the honesty of sexually-selected ornaments [32].

We test the hypothesis that the expression of orange body coloration in *A*. *sagrei* is maintained by positive effects on individual fitness. While expressing orange coloration in parts of the body other than the dewlap may increase a male’s probability of mating, we predicted that there would be costs associated with the allocation of additional pigments to the skin including: 1) reduced overall body condition, 2) reduced locomotor performance, 3) reduced immune function, and 4) increased susceptibility to predation.

## Methods

### Animal husbandry

Wild-caught *Anolis sagrei* (n = 68) were purchased from Big Apple Pet Supply (Orlando, FL, USA). Our research was approved by the Institutional Animal Care and Use Committee at Dartmouth College (IACUC; permit number 00002097-r3). All lizards were kept in individual 10-gallon glass terraria with a coarse woody substrate and a potted plant. All terraria were housed in a room with a regulated temperature of 24°-28°C. All tanks were illuminated by a UV-B bulb and were provided heat from a 40W incandescent lamp on an automated 10:14 day-night cycle. Lizards were fed *Acheta domestica* crickets every three days. Tanks were misted and plants were watered daily to control humidity. Following arrival in the laboratory, all lizards were given 4 weeks to habituate to the new conditions before any analyses were conducted. Individuals were sexed based on the presence (male) or absence (female) of enlarged post-anal scales and only adults (SVL > 34 mm) were used in this study. All individuals were weighed (nearest g), measured for snout-vent length (SVL, nearest mm), and photographed at the beginning of the experiment.

### Assignment of color categories

All lizards were assigned to one of three categories: “orange,” “brown,” and “redhead.” Both males and females of *Anolis sagrei* display bright yellow, orange, and red coloration on their dewlaps. Any males or females displaying cryptic hues such as brown, grey, or black on all parts of the body apart from the dewlap were categorized as “brown.” Some individuals of *A*. *sagrei* also exhibit bright red or orange color on the dorsal surface of the head. This coloration is characteristic of one of three dorsal pattern morphs of female *A*. *sagrei* (“Diamond”; [33]) but is also present in some males. This morphotype was categorized as “redhead.” Males and females exhibiting orange or red coloration anywhere on the body other than on the dewlap or head were considered “orange”; most individuals in this category had greater than 10% orange coverage. Redhead females exhibiting orange or red on other parts of the body were included in the “orange” category and were excluded from the “redhead” category (n = 1).

Images of the dorsal view of orange and redhead lizards were captured with a Nikon D40 digital camera with a Nikon DX AF-S Nikkor lens. Images were analyzed using ImageJ photo analysis software [34]. The individual in the image was outlined to determine the total number of pixels representing the dorsal view of the animal; limbs were excluded from the outline. Areas on the animal containing red or orange pigmentation were outlined in the same manner. The number of orange pixels was then divided by the number of total pixels to quantify what proportion of the animal expressed orange pigmentation. This variable is hereafter referred to as “%Orange”. Individuals that did not exhibit any red or orange pigmentation were scored as 0% orange.

### Female preference tests

We measured the time that females spent associating with pairs of males to determine whether they preferred orange males over brown. Three weeks before behavioral trials, and after all physiological measurements were complete, we adjusted light cycles in all tanks to 10:14 D:L to simulate lighting conditions during the breeding season [35]. Modifying the methods outlined in Swierk et al. [35], we gave females the opportunity to associate with two different males, one brown and one orange, during 10 minute trials. Individual orange males were paired with brown males whose SVL and mass most closely resembled their own, as these two variables can affect mating success [36]. Although females were exposed to several different pairs of males, the males remained in the same male-male pair for each trial. All individuals were placed in a plexiglass arena divided into three compartments, constructed so that the female could see both males, but neither male could see the other. Compartments were illuminated from above with a 40W incandescent lamp.

Prior to the onset of preference trials, individuals were placed under an opaque plastic cup to acclimate for 10 min. After the acclimation period, we removed the cup and observed the behavior of the three lizards from behind a blind, recording all interactions to digital video over the course of 10 minutes. We recorded the number of head-bob and dewlap displays performed by each male as well as the number of head-bobs directed by the female toward each male because female head-bobs are a signal of sexual receptivity [37]. As another measure of preference, the total time the female spent associating with each male in each 10 minute trial was recorded. Females were considered to be associated with a particular male if she was on his side of the arena, and either the male or female was displaying continuously. A display followed by another display within 10 seconds was considered continuous.

### Color assays

Seven individuals died (of natural causes) during the course of our study. These were frozen immediately and used to characterize the pigment content of the skin. We modified the methods outlined in McGraw et al. [38]. Dorsum and dewlap tissue were collected from orange (n = 3) and brown (n = 4) males and washed to remove any blood contamination, as pigments circulating in the blood could confound results. Tissue was chopped finely and placed in a glass vial with a 1:1 (*v/v*) mixture of non-aqueous hexane:tert-butyl methyl ether (TBME). Vials were heated in a water bath at 50°C for 1.5 hours. In a separate vial, one drop of concentrated HCl (12 N) was added to 25ml of pyridine. This solution was mixed with distilled water (1:2, *v/v*), and then added to the hexane:TBME containing the tissue sample (1:2:1, HCl/pyridine:water:hexane/TBME). Vials were maintained in the dark overnight to allow the hexane:TBME phase to separate from the aqueous pyridine phase. Lipid-soluble carotenoids bind to the hexane:TBME top phase while water-soluble pigments, such as pterins, are present in the aqueous pyridine bottom phase.

To compare the relative pigment concentrations and to construct visual models, we measured the reflectance of each live lizard using an Ocean Optics Jaz spectrometer with a bifurcated full-spectrum light source probe fitted with a custom 45° angle cover. Reflectance spectra were taken from the side of the body of each lizard, the most “orange” portion of the skin of each orange lizard, the head of redhead females, and the dewlaps of all males. Additionally, we collected reflectance spectra of brown bark and green leaves to construct the visual discrimination models using realistic background colors.

### Physiological measurements

To compare the immune responses of orange and brown lizards, we used methods for *Anolis* outlined in Calsbeek et al. [33]. Briefly, we injected 0.1mg of a mitogen, phytohaemagglutinin (PHA-P), dissolved in 0.01mL of phosphate-buffered saline (PBS) in the left footpad of each individual (males and females). The same volume of PBS alone was injected into the right footpad as a control. Footpad depth was measured three consecutive times to the nearest 0.1mm before injection, and 24 hours after injection. We averaged the consecutive measurements for use in analyses. We measured the response to the mitogen by calculating the difference in swelling before and after injection for each footpad. We then subtracted the differences for swelling in the footpad injected with PHA-P and swelling in the footpad injected with PBS alone for each lizard. We assumed that a greater difference in swelling between the left and right footpads was indicative of a stronger immune response.

We used the residuals from a linear regression of snout-vent length (SVL) and mass as a measure of corpulence. Corpulence, a measure of body condition, has been shown to correlate positively with overall health in reptiles [24]. To measure sprinting speed, individuals were removed from their enclosures and given 10 minutes to rest in a small burlap bag at 24–28°C. Lizards were then chased along a one-meter wooden dowel that was inclined at 40 degrees. The trial ended when the lizard sprinted the full length of the dowel three individual times. Trials were recorded using a Canon VIXIA HF S200 video camera. Digitized videos were analyzed frame-by-frame using Avidemux 2.6 opensource software. For each trial, we calculated the fastest speed at which the lizard ran a 10 cm length on the dowel. The fastest speeds from each of the three trials were averaged to calculate a final sprint speed. To measure endurance, individuals were removed from their enclosures and given 10 minutes to rest in a small burlap bag at 24–28°C. They were then run on a custom-built treadmill rotating at 0.6 km/hr until exhaustion, measured as a loss of the righting response. Time to exhaustion in seconds (endurance time) was recorded as a measure of endurance [39]. Lizards were given a minimum of 24 hours to rest between the sprinting speed and endurance tests.

### Visual models

We constructed receptor-noise discrimination models [40] to calculate the color distances of each *A*. *sagrei* color category as viewed by violet- and ultraviolet-sensitive birds. We additionally modeled all *A*. *sagrei* color categories as viewed by conspecifics. All models were constructed using the “pavo” package, a framework for analyzing color using spectral data [41] in the R Statistical Programming language [42]. We first calculated quantum catches for each photoreceptor type. We used the average violet-sensitive and ultraviolet-sensitive bird spectral sensitivities included in the package. These categories are biologically relevant because bird vision is highly conserved and *Anolis* are preyed upon by multiple avian species [30, 31]. We used the optical transmission spectrum of the blue tit [43] and the irradiance spectra of standard daylight (D65) and forest shade. We tested the optical transmission spectrum of a blackbird as well, but did not find any qualitative differences, so we do not report those values here. *Anolis sagrei* spectral sensitivities were obtained from Fleishman et al. [44]. We then estimated the ΔS–the contrast between each color of *Anolis* and background colors–in units of ‘just noticeable differences’ (JNDs). A JND of 1 is the threshold at which, under ideal conditions, two colors are easily discriminable. Values less than 1 are not discriminable, and values greater than 1 become increasingly discriminable. Typically, values greater than 2 are considered easily discriminable. Relative photoreceptor abundances were obtained from [45] for birds and from ([44]; 1:1:1:1) for *Anolis*. To choose the photoreceptor abundances for birds, we used the average values reported for Pelicaniformes for the violet-sensitive birds, as herons and egrets are predators of *Anolis* where they co-occur [46]. We used the average reported values for Passeriformes for ultraviolet-sensitive birds, as grackles and thrashers are also predators of *Anolis* [30, 46]. The Weber fraction for lizards is not known but was assumed to be *w* = 0.1 as in [44]. This value is based on known Weber fractions in birds and so was also used for both bird visual systems. We measured the JNDs of each morphotype of lizard and of male dewlaps using multiple reflectance spectra as viewed by both violet-sensitive and ultraviolet-sensitive birds on a brown bark or green leafy background, in both forest shade and idealized daylight (D65).

### Statistical analyses

All statistical analyses were conducted in JMP v.13.0.0 unless otherwise noted. All variables conformed to the assumptions for using parametric statistical analyses. To test the hypothesis that orange coloration comes at a physiological cost to lizards, we compared the corpulence, immune response, sprint speed, and endurance of lizards from each color category. We compared the corpulence measurements of brown, orange, and redhead lizards using an ANOVA. We used a one-way ANOVA to compare the immune responses of brown, orange, and redhead lizards. If the ANOVAs indicated a significant difference, we used a post-hoc Tukey- Kramer HSD test to identify differences between pairs of color categories. We also tested for a relationship between immune response and %Orange using a least squares regression. We controlled for variation in the size of individual lizards when conducting this analysis by including SVL as a covariate.

We used a Student’s t-test to compare the mean sprint speed of orange and brown lizards (redhead lizards were excluded from this analysis). We used a least squares regression to test for a relationship between sprint speed and %Orange. We used a one-way ANOVA to compare the mean endurance time of orange, brown, and redhead lizards. We used a least squares regression to test for a relationship between endurance time and %Orange.

To test the hypothesis that females would prefer one color variant of males over another, we recorded two measures of female preference: the difference in numbers of head-bobs directed at orange vs. brown males (ΔFH) and the difference in time spent associating with each male (ΔTime; brown-orange). To assess whether females performed more head-bobs or spent more time with a male based on color, we used a one sample t-test for ΔFH and ΔTime, testing whether the mean of each distribution was significantly different from zero. We used a least squares regression to identify relationships between the measures of female choice, ΔMH (the difference in number of head-bobs performed by the orange and brown males in each trial), and ΔMD (the difference in number of dewlap flashes performed by the orange and brown males in each trial).

Reflectance spectra were collated into 1nm bins and negative values were added to the absolute minimum value to normalize and reduce noise. We used the R package “pavo” [41] to calculate the red chroma of each reflectance spectrum. Red chroma refers to the relative contribution of the “red” spectral range (605–700 nm) to the total brightness. The concentration of both carotenoids and pterins in *A*. *sagrei* dewlap skin correlates to red chroma [47, 48]. Therefore, it is possible to compare the relative concentration of orange pigments in orange, brown, redhead, and dewlap skin. We used ANOVA to compare the red chroma of brown skin, orange skin, red heads, and dewlaps.

## Results

Based on the two measures of female preference for males (ΔFH and ΔTime), females did not show a preference for orange or brown males (Table 1). Females spent a significantly greater amount of time with males who performed more head-bobs than their counterparts (Table 1) but this pattern was not driven by difference in male body coloration. The same pattern did not hold for dewlap flashes statistically accounting for variation in body mass. Females performed more head-bobs at males who performed more dewlap displays and more head-bob displays relative to their male counterparts (Table 1).

**Table 1:** Effects of male behavior and color on female preference.

ΔTime				
	MH*	R^2^ = 0.36	*F*_2,19_ = 5.33	*P* = 0.015
	MD*	R^2^ = 0.16	*F*_2,19_ = 1.8	*P* = 0.19
	MC	—	*T*_21_ = 0.16	*P* = 0.88
ΔFH				
	MH	R^2^ = 0.48	*F*_1,20_ = 18.4	*P* = 0.0004
	MD	R^2^ = 0.29	*F*_1,20_ = 8.2	*P* = 0.0096
	MC	—	*T*_21_ = 1.15	*P* = 0.26

Results of female choice experiment in which female lizards were presented with one brown and one orange male. The difference in the amount of time spent with the brown and orange male (ΔTime) and the difference in the number of head-bobs performed at each male (ΔFH) are measures of female preference. MH = number of male head-bobs directed towards the female. MD = number of male dewlaps directed towards the female. MC = male color (brown or orange). Asterisks (*) indicate values that were controlled for mass.

Pigments extracted from the dewlaps of orange and brown lizards separated into both the water- and lipid-soluble phases, suggesting that both carotenoids and pterins are present in this tissue. However, the pigments extracted from three of four brown dorsa and all orange dorsa were found only in the bottom phase, indicating that pterins, but not carotenoids, were present. One brown individual displayed neither carotenoids nor pterins in its dorsum tissue despite having both in its dewlap.

An ANOVA indicated a significant difference in red chroma among brown skin, orange skin, red heads, and dewlaps (*F*_3, 64_ = 95.3, *P* < 0.0001). The red chroma of all pairs differed significantly from one another in the post-hoc Tukey-Kramer HSD except redhead-orange (*P* = 0.67).

**Fig 1:**
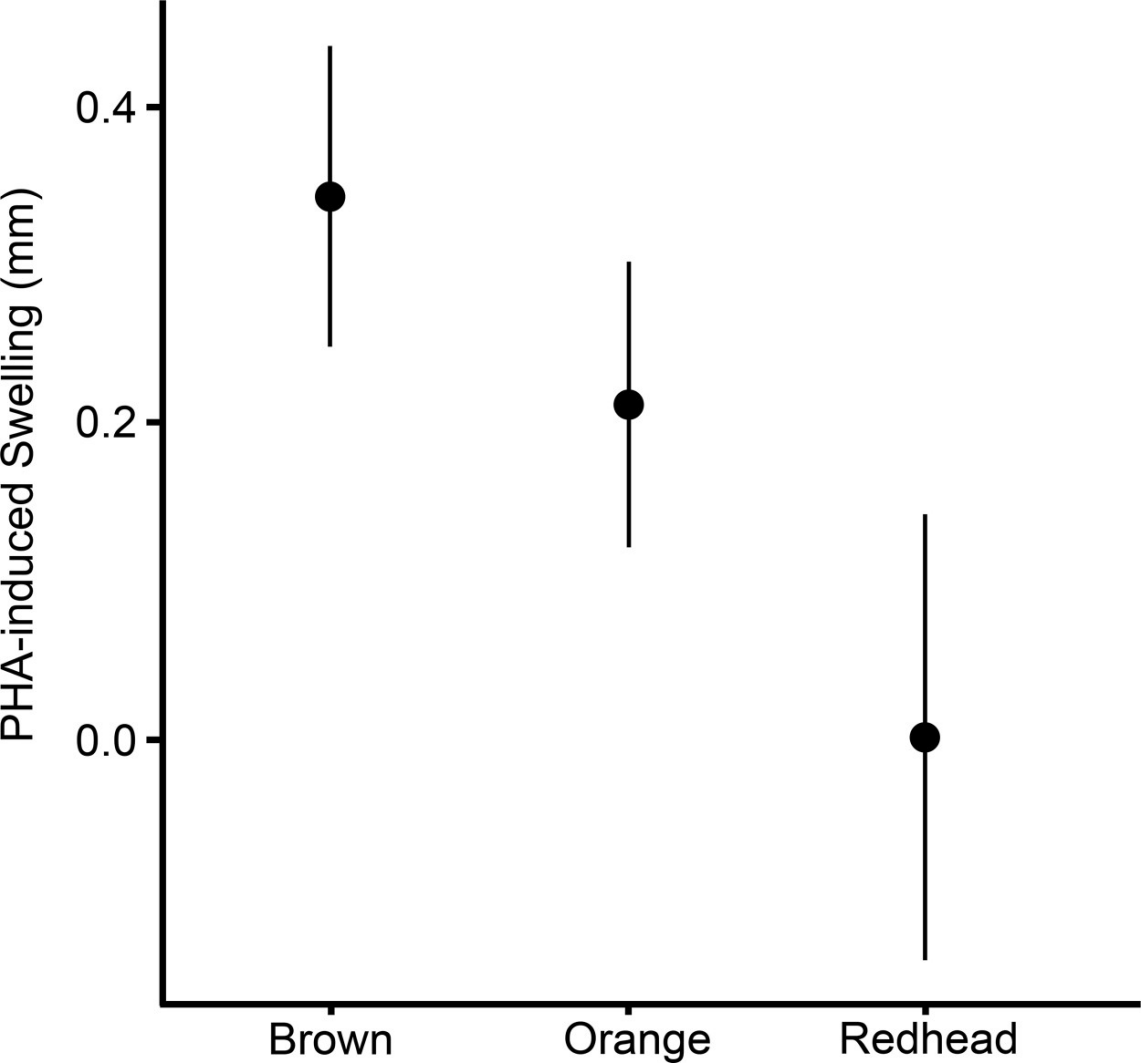
The immunocompetence of orange lizards (n = 12) did not differ significantly from that of brown (n = 25) or redhead lizards (n = 5). Brown lizards and redhead lizards differed significantly in immunocompetence. Error bars represent two standard errors from the mean (Means: Brown = 0.34, Orange = 0.24, Redhead = 0.0005).

There was a significant difference in the amount of swelling due to PHA injection among brown, orange, and redhead lizards (ANOVA *F*_2, 46_ = 6.70, *P* = 0.003) (Fig 1). A post-hoc Tukey-Kramer HSD indicated a significant difference between brown and redhead lizards (*P* = 0.0009). No significant difference in immune response was observed between orange and brown lizards or orange and redhead lizards. Redhead lizards exhibited nearly no response to PHA (Mean = 0.0005mm). Variation in immunocompetence was not associated with sex, mass, SVL, or %Orange (Table 2).

**Table 2:** Fitness measures assessed in this study and their relationships to individual attributes.

PHA Swelling				
	Mass	R^2^ = 0.0060	*F*_1,36_ = 0.20	*P* = 0.66
	SVL	R^2^ = 0.032	*F*_1,40_ = 1.3	*P* = 0.26
	%Orange	R^2^ = 0.052	*F*_1,45_ = 2.5	*P* = 0.12
	Sex	—	*T*_40.2_ = 0.71	*P* = 0.48
Sprint Speed				
	Mass	R^2^ = 0.11	*F*_1,21_ = 2.6	*P* = 0.12
	SVL	R^2^ = 0.12	*F*_1,22_ = 3.0	*P* = 0.099
	%Orange	R^2^ = 0.0095	*F*_1,20_ = 0.19	*P* = 0.67
	Sex	—	*T*_12.9_ = -0.57	*P* = 0.58
Endurance				
	Mass	R^2^ = 0.042	*F*_1,37_ = 1.6	*P* = 0.21
	SVL	R^2^ = 0.016	*F*_1,39_ = 0.64	*P* = 0.43
	%Orange	R^2^ = 0.013	*F*_1,39_ = 0.51	*P* = 0.48
	Sex	—	*T*_38.0_ = 1.5	*P* = 0.15

Corpulence did not differ significantly among brown, orange, and redhead lizards (ANOVA *F*_2, 48_ = 0.085, *P* = 0.92). Variation in lizard sprint speed was not correlated with differences in body coloration (Brown and Orange; student’s *t*_21.9_ = -1.34, *P* = 0.19). In addition, no relationship was observed between sprint speed and %Orange (Table 2). We observed no relationship between sprint speed and sex, mass, or SVL (Table 2). Lizards did not differ in running endurance as a function of their body color (ANOVA *F*_2, 38_ = 0.059, *P* = 0.94). Endurance was not influenced by %Orange, sex, mass, or SVL (Table 2). The summary statistics of all traits measured in this study are presented in Table 3.

**Table 3:** The means and standard deviations of traits measured in this study. Presented as: mean (N, SD).

	Trait	Brown	Orange	Redhead
	% Orange	0	69.6 (11, 0.36)	17.1 (9, 0.07)
Female				
Preference Test	% Difference in SVL between paired males	1.54 (5, 1.4)		-
	Time spent with male (s)	104 (23, 133)	86.4 (23, 98.1)	-
	Number of headbobs by male	32.0 (23, 43.0)	29.7 (23, 32.0)	-
	Number of dewlaps by male	1.21 (23, 2.26)	5.52 (23, 12.5)	-
	Number of headbobs by female	11.8 (23, 15.4)	13.8 (23, 15.2)	-
Physiological Measures				
	Δ PHA (mm)	0.34 (29, 0.26)	0.21 (11, 0.16)	0.002 (5, 0.20)
	Corpulence	0.21 (28, 3.18)	0.21 (15, 3.42)	0.02 (8, 3.36)
	Sprint Speed (10cm/s)	0.16 (11, 0.04)	0.14 (12, 0.04)	-
	Endurance (s)	71.3 (22, 23.4)	68.6 (8, 15.5)	70.2 (7, 25.7)

Visual modeling results suggest that orange lizards are never easily discriminable (ΔS > 2) by ultraviolet- or violet- sensitive predators, regardless of background or lighting condition (Table 4). They were equally inconpicuous to their conspecifics (Tables 4 and 5). The red heads of redhead lizards were rarely easily discriminable by UV-sensitive birds and conspecifics. They were never easily discriminable by violet-sensitive birds, regardless of background or light environment (Table 4). Dewlaps were always more often easily discriminable than orange, brown, or redhead patches, regardless of viewer, background, or light environment (Table 4).

**Table 4:** 

*A*. *sagrei*		UV Bird		V Bird		*A*. *sagrei*	
Color Category	Background Color	Daylight	Shade	Daylight	Shade	Daylight	Shade
Brown (n = 31)	Brown	9.7	9.7	3.2	3.2	12.9	19.4
	Green	9.7	9.7	3.2	3.2	9.7	9.7
Orange (n = 24)	Brown	0	0	0	0	0	4.2
	Green	0	0	0	0	0	0
Redhead (n = 11)	Brown	18.2	18.2	0	0	18.2	18.2
	Green	9.1	9.1	0	0	9.1	9.1
Dewlap (n = 22)	Brown	59.1	59.1	59.1	59.1	59.1	59.1
	Green	50.0	50.0	22.7	27.3	50.0	50.0

Percent of spectra with ΔS > 2 for each color category of *Anolis sagrei* as viewed by a generic ultraviolet-sensitive bird, a generic violet-sensitive bird, and a conspecific on brown bark or green leaves, in daylight and shade. JNDs are measured in chromatic distance (ΔS), where ΔS = 1 is the threshold of discrimination under idealized situations, with larger values becoming more discriminable and ΔS<1 indistinguishable. We assume here that ΔS>2 are likely discriminable.

**Table 5:** 

*A*. *sagrei*		UV Bird		V Bird		*A*. *sagrei*	
Color Category	Background Color	Daylight	Shade	Daylight	Shade	Daylight	Shade
Brown (n = 31)	Brown	48.4	45.2	90.3	90.3	41.9	38.7
	Green	77.4	77.4	41.9	61.3	77.4	67.7
Orange (n = 24)	Brown	70.8	70.8	95.8	95.8	66.7	66.7
	Green	91.7	91.7	37.5	41.7	75.0	66.7
Redhead (n = 11)	Brown	63.6	63.6	81.8	81.8	63.6	63.6
	Green	72.7	72.7	36.4	36.4	72.7	63.6
Dewlaps (n = 22)	Brown	27.3	27.3	18.2	18.2	27.3	27.3
	Green	36.4	36.4	27.3	22.7	40.9	36.4

Percent of spectra with ΔS < 1 for each color category of *Anolis sagrei* as viewed by a generic ultraviolet-sensitive bird, a generic violet-sensitive bird, and a conspecific on brown bark or green leaves, in daylight and shade. JNDs are measured in chromatic distance (ΔS), where ΔS = 1 is the threshold of discrimination under idealized situations, with larger values becoming more discriminable and ΔS<1 indistinguishable. We assume here that ΔS>2 are likely discriminable.

On brown backgrounds, the percentage of individuals with a ΔS < 1 (1 being the threshhold for likely discriminability) was higher for orange lizards than brown lizards in both lighting conditions and as seen by all tested visual systems (Table 5). On green backgrounds, the percentage of likely indiscriminabile lizards was higher for orange than brown lizards when viewed by UV-sensitive birds (Table 5). In all other cases, orange lizards were less discriminable than or as discriminable as brown lizards.

## Discussion

**Fig 2:**
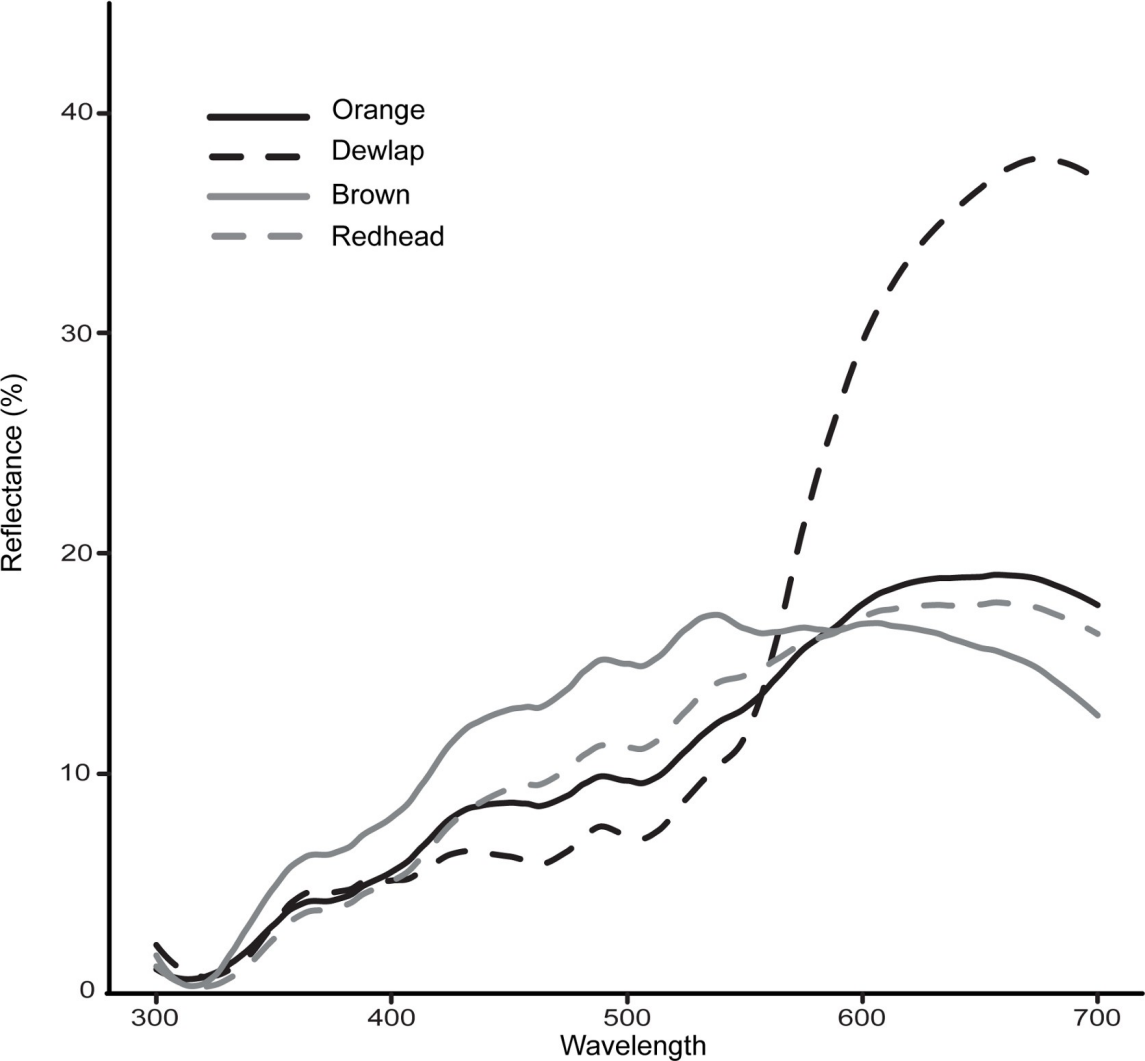
Despite perceived similarities to human observers, the reflectance spectrum of orange skin is much more similar to brown and redhead coloration than dewlap coloration. Note especially the red spectrum (605-700nm). Orange and brown lizards differed significantly in their red chroma, and thus, their pigment concentration. Orange and redhead patches did not differ significantly in red chroma and may thus have similar pigment concentrations. Reflectance curves were smoothed for presentation after all statistical analyses were conducted.

Based on the results of this study, orange *A*. *sagrei* do not experience a sexually selected advantage attributable to their body coloration. Females did not respond differentially to orange coloration despite the fact that they are predisposed to respond to orange coloration in the dewlap. This may be because the “orange” expressed in the body and the “orange” expressed in the dewlap exhibit very different spectral characteristics (Fig 2). Our data suggest that these colors may be dissimilar when perceived by *A*. *sagrei*. Additionally, while there may be costs associated with orange color in *A*. *sagrei*, they are unlikely to be related to physiological or immunological function. Taken together, our results provide no evidence that orange coloration outside of the dewlap is an honest signal of sender quality.

Female preference was more readily explained by the relative activity levels of the males, as measured by the number of dewlap flashes and head-bobs a male performed towards a female, rather than variation in male body coloration. Our results are similar to what Greenberg and Noble [49] observed in *Anolis carolinensis*; males that courted females more vigorously and more frequently were more successful at attracting females. It has been suggested that head-bob frequency is an honest signal of physical stamina [50]. After our main analyses were conducted, we compared the number of head-bobs and dewlaps performed by each male in our study and found that endurance time was significantly positively related to both behaviors. A comparison of the activity levels of brown and orange males showed that there was no difference in the number of dewlap flashes or head-bobs performed by orange and brown males, again suggesting that color does not affect physical fitness.

As *Anolis* dewlaps are known to contain both carotenoids and pterins [25, 51], we used dewlap tissue as a positive control in our color assays. Extractions revealed no evidence of carotenoids in the dorsum skin of either orange or brown individuals. However, both carotenoids and pterins were present in the dewlap skin, indicating that the color assays worked appropriately. The absence of carotenoids in the dorsa of all seven individuals that we sampled points to the absence of this pigment in the dorsal coloration of *Anolis sagrei*. Supporting this conclusion, [52] found that carotenoid supplementation did not change the dorsum coloration of female *A*. *sagrei*. While McGraw et al. [38] report the presence of carotenoids in *Anolis* dorsa, their data come from another species of anole (*Anolis carolinensis*) that displays bright green coloration throughout the body which is due to a combination of structural color and carotenoid pigment.

We found pterin pigments in the dorsa of both brown and orange individuals, which suggests that the difference in coloration may not simply be due to the presence or absence of these pigments. As in our study, Cuervo et al. [48] found pterins in lizard skin (*Acanthodactylus erythrurus*) that both did and did not appear orange. They hypothesize that melanin may conceal the presence of other pigments. Other explanations include changes to integument nanostructure and to the distribution of pterins within chromatophores [48].

Because the red chroma of all the pairwise comparisons of color categories differed significantly with the exception of redhead-orange, it is likely that the orange skin of lizards has a different concentration of pterins than that of brown skin and dewlaps (Fig 2) and that orange skin has a concentration of pterins comparable to that of the redhead phenotype.

Driessens et al. [24] found that both males and females with brighter dewlaps (and thus lower carotenoid concentrations) experienced a smaller swelling response to PHA-P. They argued that lizards that allocated more carotenoids to immune function were able to mitigate the effects of the mitogen more easily than those that allocated more carotenoids to signaling function. We expected a similar pattern in swelling response to PHA-P, but orange and brown lizards displayed nearly identical immune responses. The relationship between dewlap carotenoid concentration and immunocompetence found by Driessens et al. [24], may not be generalizable to dermal pterin concentration and immunocompetence.

Immune function has been shown to correspond to several different phenotypic traits in *A*. *sagrei*. For example, Calsbeek et al. [33] observed differential response to a novel mitogen (phytohemagglutanin; PHA) in the three dorsal pattern morphs of female *A*. *sagrei*. Whereas Calsbeek et al. [33] identified discreet female morphs (i.e., Diamond, Diamond-bar, and Bar), our study used the presence or absence of a red head (which included both males and females) to identify a potential intermediate between orange and brown morphotypes. The redhead category in this study is most comparable to the “Diamond” morph of female *A*. *sagrei* in the Calsbeek et al. [33] study characterized by a diamond-like dorsal pattern and frequently featuring a red head. While they found that the immunocompetence of “Diamond-bar” females differed significantly from those of both “Diamond” and “Bar” females, they found no significant difference in immunocompetence between “Diamond” females and “Bar” females. Because we found no difference in immunocompetence between sexes, the effect of males is likely negligible. Calsbeek et al. [33] suggest that immune function may be genetically correlated to morphotype. Because we saw no effect of orange skin on immune function, genetic correlation may serve to explain the difference observed between brown and redhead lizards. As females can be both orange *and* express discrete dorsal patterning phenotypes, it may be informative to compare the immunocompetence of orange and non-orange females while also categorizing based on dorsal patterning. A study of this design would be more likely to isolate the effects of full-bodied orange coloration on immune function.

Color did not have a significant effect on overall body condition, suggesting that orange, brown, and redhead lizards do not differ in this respect. Additionally, color was not related to endurance or sprint speed in *A*. *sagrei*. Similar studies found that antioxidant supplements did not enhance physical endurance in humans (Vitamin C: [53–55]; Vitamin E: [55–57]; Antioxidant blends: [55, 58]) or in rats [16]. While antioxidants may mitigate damage done to tissues by free radicals, they may not improve performance *per se*. That is, color may affect how antioxidants are allocated, but is not likely related to physical performance in *A*. *sagrei*.

It is likely that predators of *A*. *sagrei* cannot easily distinguish orange individuals from bark or leaves. Despite appearing conspicuous to a human visual system, visual models suggest that orange body colors are less conspicuous than dewlaps, which must be conspicuous to successfully function as signals (Tables 3 and 4). The large differences between orange skin and dewlaps in the red portion of the visual spectrum (Fig 2) may explain why *A*. *sagrei* and their predators perceive them as disparate colors. Because orange skin was less conspicuous than even brown skin, orange individuals may be less easily detected by predators.

Our study is the first to document the orange phenotype found in Floridian populations of *A*. *sagrei*. Despite significant differences in pigment concentration in the skin, orange lizards were effectively identical in immunocompetence and physical ability to brown members of the same species. Additionally, orange and brown males did not differ in their ability to attract mates. Our study suggests that the maintenance of the novel phenotype could be due to enhanced predator avoidance. The effect of an over-dominant allele or of genetic drift may also be considered. Using this phenotype, future studies may treat *A*. *sagrei* as a model to identify how novel traits proliferate through and among populations in real time.

## Supporting information

S1 Table JNDs (ΔS) of *Anolis sagrei* on different surfaces in a D65 illuminant as viewed by an ultraviolet-sensitive bird, a violet-sensitive bird, and a conspecific lizard. (DOCX)

S2 Table JNDs (ΔS) of *Anolis sagrei* on different surfaces in a forest shade illuminant as viewed by an ultraviolet-sensitive bird, a violet-sensitive bird, and a conspecific lizard. (DOCX)

S3 Table SVL and mass of male pairs used in female preference trials. Range of difference, mean difference, and standard deviation is also presented. Numbers associated with males are those assigned to them throughout all experiments.
(DOCX)

S1 Dataset Compiled data from the traits presented in Table 3. (XLSX)

S2 Dataset The spectral outputs used to calculate JNDs (ΔS) and red chroma. (XLSX)
