# Supplementary material for: A novel body coloration phenotype in Anolis sagrei: Implications for physiology, fitness, and predation
Source: PLoS One. 2018 Dec 31;13(12):e0209261. doi: 10.1371/journal.pone.0209261 (PMC6312277; doi:10.1371/journal.pone.0209261)
Supplement: S1 Table — (DOCX) [file pone.0209261.s001.docx]

S1 Table. JNDs (ΔS) of *Anolis sagrei* on different surfaces in a D65 illuminant as viewed by an ultraviolet-sensitive bird, a violet-sensitive bird, and a conspecific lizard.

|  | Ultraviolet Bird | | Violet Bird | | Lizard | |
| --- | --- | --- | --- | --- | --- | --- |
|  | Brown Bark | Green Leaf | Brown Bark | Green Leaf | Brown Bark | Green Leaf |
| **Orange Lizards** |  |  |  |  |  |  |
| Spectrum 13 | 0.81 | 0.72 | 0.62 | 0.87 | 1.64 | 0.96 |
| Spectrum 30 | 0.11 | 0.82 | 0.13 | 1.33 | 0.11 | 1.12 |
| Spectrum 35 | 0.75 | 0.68 | 1.06 | 0.81 | 1.01 | 0.33 |
| Spectrum 36 | 0.32 | 0.61 | 0.34 | 0.97 | 0.68 | 0.51 |
| Spectrum 42 | 0.97 | 0.82 | 0.85 | 0.76 | 1.77 | 0.99 |
| Spectrum 51 | 0.50 | 0.67 | 0.28 | 1.04 | 1.00 | 0.58 |
| Spectrum 59 | 2.41 | 2.00 | 2.76 | 1.88 | 4.43 | 3.54 |
| Spectrum 64 | 0.42 | 0.67 | 0.25 | 1.08 | 0.90 | 0.59 |
| Spectrum 70 | 1.73 | 1.37 | 2.36 | 1.56 | 2.74 | 1.81 |
| **Redhead Lizards** |  |  |  |  |  |  |
| Spectrum 14 | 0.14 | 0.69 | 0.16 | 1.13 | 0.32 | 0.75 |
| Spectrum 16 | 0.23 | 0.66 | 0.24 | 1.07 | 0.48 | 0.62 |
| Spectrum 42 | 0.97 | 0.82 | 0.85 | 0.76 | 1.77 | 0.99 |
| Spectrum 44 | 0.72 | 0.70 | 0.55 | 0.86 | 1.37 | 0.69 |
| Spectrum 63 | 1.60 | 1.30 | 1.49 | 0.92 | 3.17 | 2.34 |
| **Brown Lizards** |  |  |  |  |  |  |
| Spectrum 1 | 0.55 | 0.67 | 0.32 | 1.01 | 1.10 | 0.61 |
| Spectrum 2 | 0.34 | 0.67 | 0.20 | 1.09 | 0.72 | 0.58 |
| Spectrum 5 | 0.32 | 0.64 | 0.25 | 1.05 | 0.72 | 0.55 |
| Spectrum 7 | 0.03 | 0.78 | 0.03 | 1.26 | 0.03 | 1.00 |
| Spectrum 12 | 0.02 | 0.76 | 0.04 | 1.23 | 0.02 | 1.00 |
| Spectrum 13 | 0.82 | 0.72 | 0.62 | 0.87 | 1.64 | 0.96 |
| Spectrum 15 | 0.11 | 0.71 | 0.14 | 1.14 | 0.23 | 0.81 |
| Spectrum 18 | 0.73 | 0.71 | 0.50 | 0.92 | 1.48 | 0.83 |
| Spectrum 20 | 0.74 | 0.70 | 0.51 | 0.89 | 1.48 | 0.83 |
| Spectrum 22 | 0.20 | 0.67 | 0.18 | 1.11 | 0.45 | 0.68 |
| Spectrum 24 | 0.05 | 0.77 | 0.06 | 1.25 | 0.06 | 0.98 |
| Spectrum 41 | 0.44 | 0.66 | 0.31 | 1.03 | 0.89 | 0.53 |
| Spectrum 43 | 0.64 | 0.67 | 0.42 | 0.96 | 1.26 | 0.69 |
| Spectrum 49 | 1.78 | 1.43 | 1.72 | 1.03 | 3.69 | 2.87 |
| Spectrum 53 | 1.01 | 0.82 | 0.82 | 0.79 | 2.06 | 1.32 |
| Spectrum 58 | 0.68 | 0.68 | 0.46 | 0.88 | 1.31 | 0.67 |
| Spectrum 64 | 0.42 | 0.67 | 0.25 | 1.08 | 0.90 | 0.59 |
| Spectrum 73 | 6.74 | 6.54 | 6.78 | 6.48 | 3.43 | 2.66 |
| Spectrum 74 | 0.97 | 0.79 | 0.79 | 0.80 | 1.97 | 1.24 |
| Spectrum 75 | 0.43 | 0.64 | 0.30 | 1.01 | 0.92 | 0.55 |
| Spectrum 77 | 0.79 | 0.73 | 0.56 | 0.91 | 1.58 | 0.93 |
| Spectrum 78 | 1.89 | 1.54 | 1.94 | 1.43 | 3.51 | 2.73 |
| Spectrum 81 | 0.72 | 0.72 | 0.46 | 0.94 | 1.44 | 0.82 |
| Spectrum 84 | 0.28 | 0.67 | 0.20 | 1.12 | 0.63 | 0.62 |
| Spectrum 88 | 0.61 | 0.70 | 0.35 | 1.02 | 1.20 | 0.69 |
| Spectrum 89 | 0.70 | 0.68 | 0.49 | 0.89 | 1.40 | 0.76 |
| Spectrum 91 | 0.31 | 0.67 | 0.20 | 1.11 | 0.69 | 0.60 |
| Spectrum 94 | 0.18 | 0.70 | 0.15 | 1.16 | 0.40 | 0.73 |
| Spectrum 96 | 0.96 | 0.80 | 0.77 | 0.83 | 1.97 | 1.24 |
| Spectrum 97 | 0.65 | 0.67 | 0.43 | 0.94 | 1.28 | 0.70 |
| Spectrum 99 | 0.86 | 0.75 | 0.63 | 0.83 | 1.72 | 1.01 |
