# Supplementary material for: A novel body coloration phenotype in Anolis sagrei: Implications for physiology, fitness, and predation
Source: PLoS One. 2018 Dec 31;13(12):e0209261. doi: 10.1371/journal.pone.0209261 (PMC6312277; doi:10.1371/journal.pone.0209261)
Supplement: S2 Table — (DOCX) [file pone.0209261.s002.docx]

S2 Table. JNDs (ΔS) of *Anolis sagrei* on different surfaces in a forest shade illuminant as viewed by an ultraviolet-sensitive bird, a violet-sensitive bird, and a conspecific lizard.

|  | Ultraviolet Bird | | Violet Bird | | Lizard | |
| --- | --- | --- | --- | --- | --- | --- |
|  | Brown Bark | Green Leaf | Brown Bark | Green Leaf | Brown Bark | Green Leaf |
| **Orange Lizards** |  |  |  |  |  |  |
| Spectrum 13 | 0.86 | 0.72 | 0.63 | 0.80 | 1.74 | 0.96 |
| Spectrum 30 | 0.11 | 0.81 | 0.13 | 1.28 | 0.11 | 1.17 |
| Spectrum 35 | 0.75 | 0.68 | 1.04 | 0.81 | 1.02 | 0.30 |
| Spectrum 36 | 0.33 | 0.59 | 0.35 | 0.93 | 0.72 | 0.48 |
| Spectrum 42 | 1.00 | 0.83 | 0.86 | 0.73 | 1.87 | 0.99 |
| Spectrum 51 | 0.54 | 0.66 | 0.29 | 0.98 | 1.10 | 0.54 |
| Spectrum 59 | 2.41 | 2.00 | 2.71 | 1.85 | 4.50 | 3.53 |
| Spectrum 64 | 0.45 | 0.65 | 0.27 | 1.02 | 0.98 | 0.54 |
| Spectrum 70 | 1.74 | 1.38 | 2.33 | 1.56 | 2.85 | 1.86 |
| **Redhead Lizards** |  |  |  |  |  |  |
| Spectrum 14 | 0.15 | 0.68 | 0.16 | 1.08 | 0.34 | 0.77 |
| Spectrum 16 | 0.24 | 0.65 | 0.25 | 1.02 | 0.52 | 0.62 |
| Spectrum 42 | 1.00 | 0.83 | 0.86 | 0.73 | 1.87 | 0.99 |
| Spectrum 44 | 0.76 | 0.70 | 0.56 | 0.81 | 1.47 | 0.68 |
| Spectrum 63 | 1.63 | 1.31 | 1.49 | 0.89 | 3.27 | 2.36 |
| **Brown Lizards** |  |  |  |  |  |  |
| Spectrum 1 | 0.59 | 0.66 | 0.34 | 0.95 | 1.20 | 0.58 |
| Spectrum 2 | 0.36 | 0.64 | 0.21 | 1.04 | 0.79 | 0.54 |
| Spectrum 5 | 0.34 | 0.62 | 0.27 | 1.00 | 0.78 | 0.52 |
| Spectrum 7 | 0.03 | 0.77 | 0.03 | 1.21 | 0.02 | 1.05 |
| Spectrum 12 | 0.01 | 0.76 | 0.04 | 1.18 | 0.12 | 1.05 |
| Spectrum 13 | 0.86 | 0.72 | 0.63 | 0.80 | 1.74 | 0.96 |
| Spectrum 15 | 0.11 | 0.69 | 0.14 | 1.10 | 0.24 | 0.84 |
| Spectrum 18 | 0.78 | 0.70 | 0.52 | 0.85 | 1.58 | 0.82 |
| Spectrum 20 | 0.78 | 0.70 | 0.53 | 0.82 | 1.58 | 0.82 |
| Spectrum 22 | 0.22 | 0.65 | 0.19 | 1.06 | 0.49 | 0.66 |
| Spectrum 24 | 0.05 | 0.75 | 0.06 | 1.20 | 0.06 | 1.02 |
| Spectrum 41 | 0.46 | 0.65 | 0.32 | 0.98 | 0.97 | 0.48 |
| Spectrum 43 | 0.69 | 0.66 | 0.44 | 0.89 | 1.37 | 0.68 |
| Spectrum 49 | 1.81 | 1.45 | 1.73 | 1.00 | 3.79 | 2.89 |
| Spectrum 53 | 1.06 | 0.83 | 0.84 | 0.71 | 2.17 | 1.33 |
| Spectrum 58 | 0.72 | 0.67 | 0.47 | 0.82 | 1.41 | 0.66 |
| Spectrum 64 | 0.45 | 0.65 | 0.27 | 1.02 | 0.98 | 0.54 |
| Spectrum 73 | 6.64 | 6.42 | 6.67 | 6.33 | 3.55 | 2.69 |
| Spectrum 74 | 1.02 | 0.80 | 0.81 | 0.73 | 2.09 | 1.25 |
| Spectrum 75 | 0.46 | 0.62 | 0.32 | 0.96 | 1.00 | 0.51 |
| Spectrum 77 | 0.83 | 0.73 | 0.58 | 0.84 | 1.68 | 0.92 |
| Spectrum 78 | 1.92 | 1.55 | 1.96 | 1.37 | 3.62 | 2.74 |
| Spectrum 81 | 0.76 | 0.71 | 0.47 | 0.88 | 1.54 | 0.81 |
| Spectrum 84 | 0.30 | 0.65 | 0.21 | 1.06 | 0.69 | 0.58 |
| Spectrum 88 | 0.65 | 0.68 | 0.36 | 0.95 | 1.30 | 0.66 |
| Spectrum 89 | 0.75 | 0.67 | 0.51 | 0.82 | 1.50 | 0.75 |
| Spectrum 91 | 0.33 | 0.65 | 0.21 | 1.06 | 0.75 | 0.56 |
| Spectrum 94 | 0.19 | 0.68 | 0.16 | 1.10 | 0.43 | 0.72 |
| Spectrum 96 | 1.00 | 0.80 | 0.79 | 0.75 | 2.07 | 1.25 |
| Spectrum 97 | 0.69 | 0.66 | 0.45 | 0.87 | 1.39 | 0.68 |
| Spectrum 99 | 0.90 | 0.75 | 0.65 | 0.76 | 1.82 | 1.01 |
