# Supplementary material for: A novel body coloration phenotype in Anolis sagrei: Implications for physiology, fitness, and predation
Source: PLoS One. 2018 Dec 31;13(12):e0209261. doi: 10.1371/journal.pone.0209261 (PMC6312277; doi:10.1371/journal.pone.0209261)
Supplement: S3 Table — Range of difference, mean difference, and standard deviation is also presented. Numbers associated with males are those assigned to them throughout all experiments. (DOCX) [file pone.0209261.s003.docx]

S3 Table. SVL and mass of male pairs used in female preference trials. Range of difference, mean difference, and standard deviation is also presented. Numbers associated with males are those assigned to them throughout all experiments.

| Male Pair | SVL (mm) | Mass (g) | %Δ SVL | %Δ Mass |
| --- | --- | --- | --- | --- |
| 10 (orange) | 53.3 | 5.53 |  |  |
| 209 (brown) | 53.4 | 5.59 | 0.18744142 | 1.07913669 |
| 30 (orange) | 54.6 | 7.33 |  |  |
| 86-1 (brown) | 55.2 | 7.40 | 1.09289617 | 0.95044128 |
| 27 (orange) | 55.7 | 5.35 |  |  |
| 38 (brown) | 55.9 | 5.79 | 0.35842294 | 7.8994614 |
| 54 (orange) | 54.8 | 6.35 |  |  |
| 59 (brown) | 53.1 | 6.40 | 3.1510658 | 0.78431373 |
| 203 (orange) | 55.5 | 5.08 |  |  |
| 46 (brown) | 53.9 | 5.18 | 2.9250457 | 1.94931774 |
|  |  |  |  |  |
